# Differential Diagnosis of Type 1 and Type 2 Papillary Renal Cell Carcinoma Based on Enhanced CT Radiomics Nomogram

**DOI:** 10.3389/fonc.2022.854979

**Published:** 2022-06-03

**Authors:** Yankun Gao, Xingwei Wang, Shihui Wang, Yingying Miao, Chao Zhu, Cuiping Li, Guoquan Huang, Yan Jiang, Jianying Li, Xiaoying Zhao, Xingwang Wu

**Affiliations:** ^1^ Department of Radiology, The First Affiliated Hospital of Anhui Medical University, Hefei, China; ^2^ Department of Radiology, The First Affiliated Hospital of Wannan Medical college, Wuhu, China; ^3^ Department of Imaging, Wuhu Second People’s Hospital, Wuhu, China; ^4^ Department of Pathology, The First Affiliated Hospital of Anhui Medical University, Hefei, China; ^5^ CT Research Center, GE Healthcare China, Shanghai, China

**Keywords:** radiomics nomogram, papillary renal cell carcinoma, differential diagnosis, computed tomography, tumour subtypes

## Abstract

**Objectives:**

To construct a contrast-enhanced CT-based radiomics nomogram that combines clinical factors and a radiomics signature to distinguish papillary renal cell carcinoma (pRCC) type 1 from pRCC type 2 tumours.

**Methods:**

A total of 131 patients with 60 in pRCC type 1 and 71 in pRCC type 2 were enrolled and divided into training set (n=91) and testing set (n=40). Patient demographics and enhanced CT imaging characteristics were evaluated to set up a clinical factors model. A radiomics signature was constructed and radiomics score (Rad-score) was calculated by extracting radiomics features from contrast-enhanced CT images in corticomedullary phase (CMP) and nephrographic phase (NP). A radiomics nomogram was then built by incorporating the Rad-score and significant clinical factors according to multivariate logistic regression analysis. The diagnostic performance of the clinical factors model, radiomics signature and radiomics nomogram was evaluated on both the training and testing sets.

**Results:**

Three validated features were extracted from the CT images and used to construct the radiomics signature. Boundary blurring as an independent risk factor for tumours was used to build clinical factors model. The AUC value of the radiomics nomogram, which was based on the selected clinical factors and Rad-score, were 0.855 and 0.831 in the training and testing sets, respectively. The decision curves of the radiomics nomogram and radiomics signature in the training set indicated an overall net benefit over the clinical factors model.

**Conclusion:**

Radiomics nomogram combining clinical factors and radiomics signature is a non-invasive prediction method with a good prediction for pRCC type 1 tumours and type 2 tumours preoperatively and has some significance in guiding clinicians selecting subsequent treatment plans.

## Introduction

Renal cell carcinoma (RCC) is the most common malignancy of the kidney in adults, accounting for approximately 85% of renal tumours (1). Clear cell RCC (ccRCC), papillary RCC (pRCC) and chromophobe RCC (chRCC), accounting for 70-80%, 10-20% and 3-7% of RCCs, respectively (2). PRCC is the second most common subtype after ccRCC. Among the subtypes of RCC, pRCC has a higher 5-year survival rate and a better prognosis. In 1997, Delahunt et al. initially subdivided pRCC into type 1 and type 2 according to morphological and immunohistochemical characteristics (3). Typically, type 1 exhibits papillae covered by a single layer of monolayer cuboidal epithelium with a lack of cytoplasm, whereas type 2 is characterized by the presence of nuclear pseudostratification (4). Previous studies have shown that type 2 tumours tend to have a higher pathological stage, a higher nuclear grade, as patients with type 2 tumours have a worse prognosis (5–7). As type 2 tumours are more aggressive, an early and accurate diagnosis is essential. Due to the low malignancy of type 1 tumours, relatively conservative treatment options such as follow-up, ablation and partial nephrectomy are usually available in clinical practice. According to the National Comprehensive Cancer Network (NCCN) RCC guidelines, less aggressive RCC can be treated by active surveillance or partial nephrectomy. In contrast, most highly aggressive RCC patients usually undergo radical nephrectomy with consideration of adjuvant therapy. The precise preoperative differentiation between these two types of the tumours will determine different treatment options and different prognoses.

Pathological biopsy by percutaneous puncture biopsy or surgical excision is the most accurate method of identifying the pRCC subtype, but it is after all an invasive test, and we would like to be able to make a non-invasive diagnosis preoperatively. Although some studies have shown a higher heterogeneity of type 2 tumours compared to type 1 tumours on conventional computed tomography (CT) and magnetic resonance imaging (MRI) images, typically type 2 tumours are large, have blurred margins and tend to invade blood vessels and metastasize to surrounding lymph nodes (8–10). However, these two types of tumours have many overlapping imaging features on conventional CT or MRI images, and it is often difficult to distinguish subtypes of pRCC based on imaging features alone.

Radiomics is a recent emerging research approach that uses high-throughput data feature extraction algorithms to translate medical images into high-dimensional, useable quantitative image features, and it uses various algorithms for deeper analysis of the features. This method can be used not only for preoperative pathological classification and grading of the tumour, but also for the prediction of prognosis and survival rate of tumour patients (11–13). Currently, radiomics studies for RCC have been focused on the identification of the three most common subtypes of RCC (ccRCC, pRCC, chRCC) and on the nuclear grading of RCC (10, 14–18). For example, Deng et al. (14)showed that CT-based texture analysis was not only able to identify ccRCC and pRCC, but also to predict the Fuhrman grade of the tumour. Some studies have shown that CT and MRI-based texture analysis techniques can differentiate between pRCC subtypes (10, 17, 18). However, to the best of our knowledge, apart from some studies that have identified pRCC subtypes based on textural features alone, there is no study that combines radiomics features with clinical factors to make a differential diagnosis of pRCC subtypes. In our study, we quantified radiomics signature by calculating the rad-score value form contrast-enhanced CT images of each patient and attempted to build a contrast enhanced CT-based radiomics nomogram that included both rad-score and clinical factors to better discriminate between the two subtypes of pRCC.

## Materials and Methods

### Patients

The ethics review board at our hospital approved this retrospective study and patient informed consent was waived. Patients who underwent non-enhanced and contrast-enhanced CT scans from January 2013 to October 2021 at our hospital for diagnosing kidney disease were considered. Percutaneous puncture or surgical excision specimens diagnosed as pRCC type 1 or 2 were selected by searching the hospital’s picture archiving and communication system (PACS). The inclusion criteria were as follows: (1) Patients who had a definitive pathologic diagnosis of pRCC. (2) Patients with available preoperative plain and enhanced CT scans, and the image quality was satisfactory for analysis (clear image with no artifacts). (3) Patients with complete clinic-pathological data. The exclusion criteria were as follows: (1) The subtype of pRCC patients could not be determined as type 1 or type 2. (2) Patients who had a history of abdominal surgery. (3) Patients received abdominal radiotherapy or chemotherapy prior to CT scan.

### CT Image Acquisition

CT scan protocols are shown in **Table 1**. A power injector administered a 90-100-ml of nonionic contrast medium (Omnipaque, GE Healthcare or Ultravist, Bayer, Schering Pharma) into the antecubital vein at a rate of 3 mL/s. Pre-contrast CT of the abdomen was first acquired, followed by three post-contrast CT scans obtained in the corticomedullary phase (CMP, acquired 30 s after contrast injection), nephrographic phase (NP, acquired 80 s after contrast injection) and excretory phase (EP, acquired 180 s after contrast injection).

**Table 1:** CT scan protocols.

Manufacturer	Siemens	General Electric	Philips
Scanner model	Sensation 64	Discovery 750	Brilliance
Sequence	Axial	Axial	Axial
Gantry rotation time (s)	0.5	0.5	0.5
Tube voltage (kV)	120	120	120
Tube current (mA)	200	250-400	180-450
Detector collimation (mm)	64×0.6	64×0.625	64×0.625
Matrix	512×512	512×512	512×512
Pitch	1.0	1.375	1.0
Slice thickness (mm)	5	5	5
Corticomedullary phase (s)	30	30	30
Nephrographic phase (s)	80	80	80
Excretory phase (s)	180	180	180

s, second; kV, kilovolt; mA, milliampere; mm, millimetre.

### CT Characteristic Evaluation

The CT image were scrutinized by two radiologists with 4 years (reader 1, Y.G.) and 7 years (reader 2, X.W.) of diagnostic abdominal imaging experience. If there was disagreement, two radiologists needed to reach a consensus. Without knowledge of the clinicopathologic data, the two readers interpreted the following CT characteristics together: the maximum diameter of the tumour on axial CT images; shape (round or not round); location (left or right); boundary (clear or blurred boundary); calcification (present or not, “calcification was considered as high density seen during pre-enhancement CT”); necrosis (present or not, “necrosis was considered as the non-enhanced liquid area of tumour accounting for more than 50% of the tumour”); renal vein invasion (present or not, “renal vein invasion was considered as the tumour tissue in the renal vein and inferior vena cava was observed on the imaging”); lymph node metastasis (present or not, “lymph node metastasis was considered as the short-axis diameter of the perirenal and retroperitoneal lymph nodes were greater than 10mm”) (9, 19).

**Figure 1:**
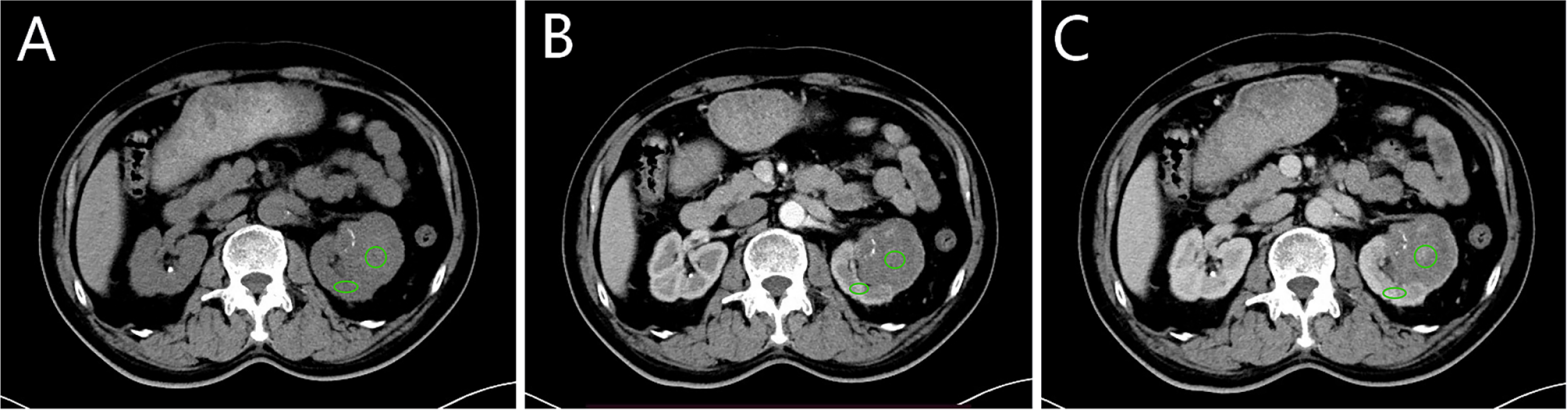
Selection of region of interests (ROIs) and reference region. **(A–C)** correspond to the non-enhanced, corticomedullary phase (CMP) and nephrographic phase (NP). The green circle is one of two tumour ROIs, selected from the parenchymal portion of the tumour where enhancement is evident. The green oval is the reference region located in the cortical portion of the kidney. The zones of ROIs and reference region are in the same position in each scan phase.

To standardize the measurement of tumour enhancement, it is generally necessary to select the appropriate region of interests (ROIs) within the tumour and characterize the tumour enhancement according to the changes in CT values of the ROIs on different scan phases. Since the tumour had been enhanced to some extent on the CMP images and the various heterogenous components of the tumour could be better displayed at the stage, all ROIs in this study were selected based on the CMP images. To accurately assess the extent of tumour enhancement, the ROIs avoided components such as necrosis, calcification, and vascularity that are clearly visible on the images and include only the substantial components of the tumour. The reader 1 select 2 non-overlapping ROIs, made separate measurements and averaged the two numbers to obtain the final measurement. Due to individual patient factors and factors that are difficult to control when performing CT scanning operations, the iodine contrast load during the scan was not identical in each case, and this variation could constitute a systematic error in the measurement of tumour enhancement. In this study, the cortical area of the kidney on the side of the tumour was selected as the reference area for iodine contrast loading normalization during the scan to correct for such systematic errors. **Figure 1** shows an example of this approach.

The ROIs selected in CMP were copied and pasted into the non-enhanced and NP images to obtain the average tumour attenuation value (TAV) in each scan phase. The average CT value of the reference area in each corresponding scan phase was used as the cortex attenuation value (CAV). The tumour enhancement value (TEV) and the cortex enhancement value (CEV) were calculated by subtracting the values of the same ROI in the non-enhanced phase: TEV_x_ = TAV_x_ – TAV_0_ and CEV_x_ = CAV_x_ - CAV_0_, where x represents the phase (0, non-enhanced; 1, CMP; 2, NP). The relative enhancement value (REV) was defined as the ratio of TEV to CEV: REV_x_ = TEV_x_/CEV_x_, representing the degree of enhancement within the tumour relative to the renal cortex (20).

### Construction of the Clinical Factors Model

Univariate analysis was used to compare the differences in clinical factors (including clinical data and CT characteristcs) between the type 1 and type 2 tumours. The significant variables acquired in the univariate analysis were used as inputs, and a multivariate logistic regression analysis was applied to establish a clinical factors model. Odds ratios (OR) was calculated for each independent factor as a relative risk estimate with a 95% confidence interval (CI).

### Three-Dimensional Segmentation of Tumour Images and Radiomics Feature Extraction

**Figure 2:**
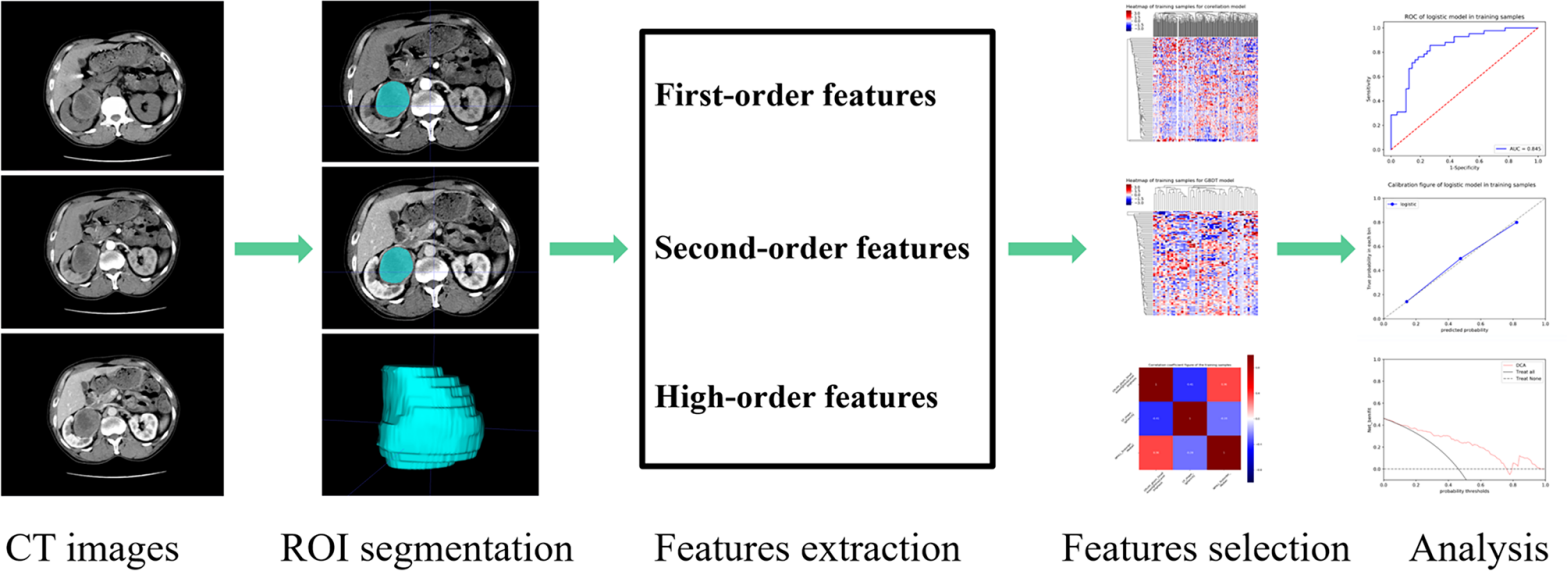
Papillary renal cell carcinoma (pRCC) study radiomics flow chart.

**Figure 3:**
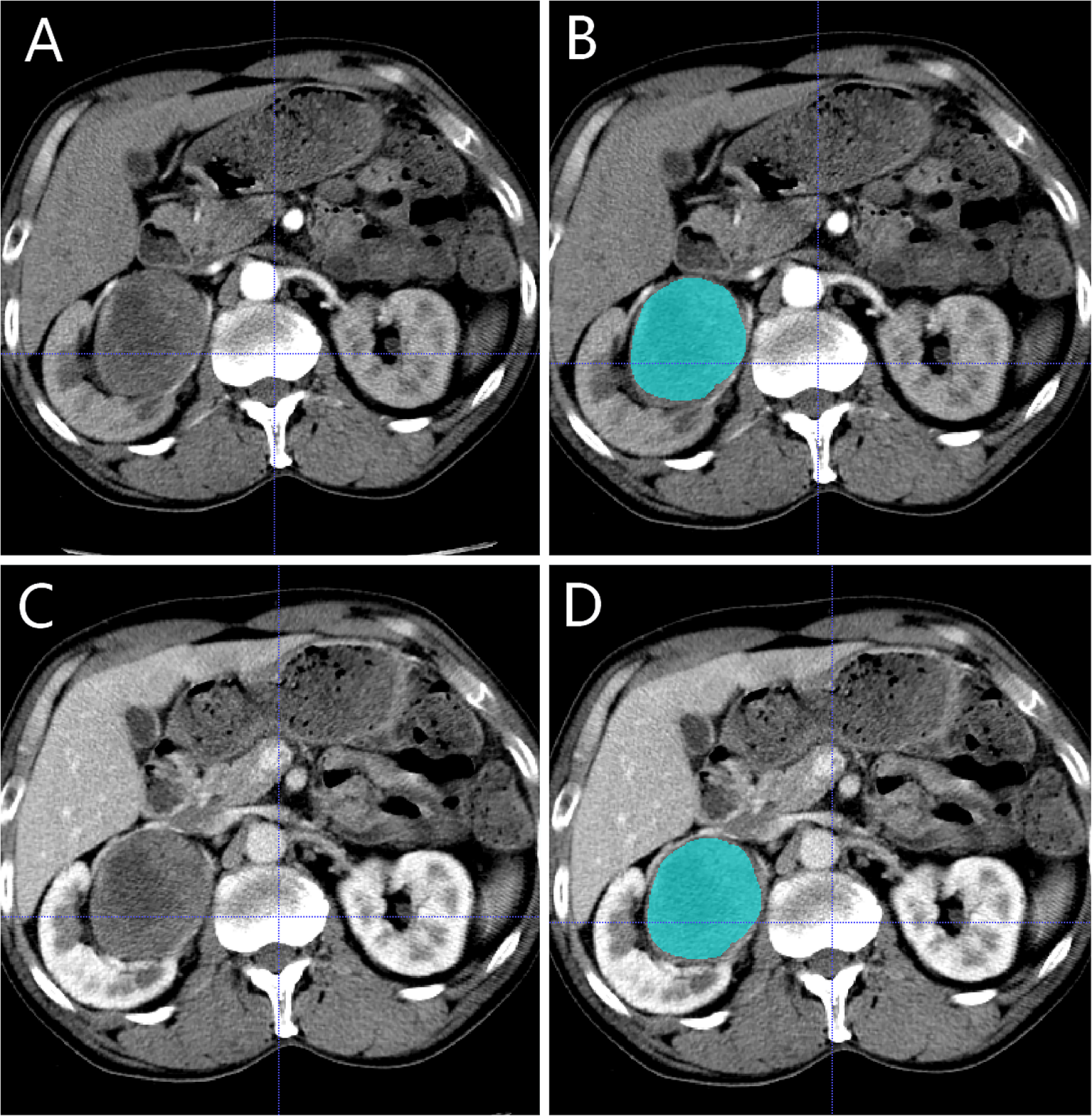
Manual segmentation of the tumour on the center axial slice of the pRCC type 2. **(A, B)** is the corticomedullary phase (CMP); **(C, D)** is the nephrographic phase (NP).

The basic steps of a radiomics model for renal tumours are detailed in **Figure 2**. Three-dimensional (3-D) segmentation of tumours was performed using the ITK-SNAP software (version 3.8, www.itksnap.org). Contouring was drawn using the ROIs within the tumour borders on CMP and NP images, 1-2mm from the tumour boundary. An example of the use of manual segmentation in a renal tumour is shown in **Figure 3**.

Features extraction was executed using the PHIgo Workstation (General Electric Company). As the images were derived from three CT scanners with different parameters, normalization and image resampling had to be performed before features could be extracted from the ROIs of the CMP and NP images. The image data is normalized using a z-score in the following form:


$$z=\frac{x-\mu }{\sigma },$$

Where *μ* is the mean of the whole data, *σ* is the standard deviation of the whole data. In addition, all CT images were resampled to 1.0×1.0×1.0 mm^3^ voxels to standardize the slice thickness use B-spline interpolation sampling technology. 1316 radiomics features were extracted from the ROIs of the CMP and NP images, respectively.

Inter-observer reliability and intra-observer repeatability of radiomics feature extraction were usually assessed using inter-and intra- class correlation coefficients (ICC). We randomly chose 20 cases of CT images (8 pRCC type 1 and 12 pRCC type 2); ROI segmentation was performed by reader 1 and reader 2. After two weeks, reader 1 repeated the same steps to evaluate the degree of matching of feature extraction. When the ICC value is more than 0.75, it indicates that the extracted features have a good consistency. Then the remaining image segmentation will be carried out by reader 1 alone.

### Construction of the Radiomics Signature

To prevent overfitting of the radiomics features, features were further selected before the construction of the radiomics signature. First, features with ICC >0.75 within the training set were retained. Second, statistically significant features were screened out using the univariate logistic analysis. Third, the most valuable features were selected using Gradient Boosting Decision Tree and multivariate logistic analysis. Finally, a radiomics score (Rad-score) was calculated by using a formula based on the radiomics features.

Rad – score was used to establish aradiomics signature multivariate logistic regression.

### Construction of Radiomics Nomogram and Performance Evaluation of Different Models

A radiomics nomogram was constructed by combining the significant variables of clinical factors and the Rad-score. Calibration curves were used to evaluate the calibration of the nomogram. The Hosmer-Lemeshow test was conducted to assess the goodness-of-fit of the nomogram. The diagnostic performance of the clinical factors model, the radiomics signature model and the radiomics nomogram for differentiating pRCC type 1 from pRCC type 2 was evaluated based on the area under the receiver operator characteristic (ROC) curve (AUC) in both the training and testing sets. To evaluate the clinical effectiveness of the radiomics nomogram, a decision curve analysis (DCA) was performed by calculating the net benefit of a threshold probability range across the training and testing sets.

### Statistical Analysis

Statistical tests were performed using SPSS (version 25.0, IBM) and IPM statistical (version 2.4.0, General Electric Company). Univariate analysis was used to compare the differences in clinical factors between type 1 and type 2 tumours. Chi-square test or Fisher exact test was used for categorical variables, and Mann-Whitney U test was used for continuous variables. A two-side *p* < 0.05 was considered significant.

## Results

### Clinical Factors of the Patients and Construction of the Clinical Factor Model

A total of 131 patients were finally enrolled in this study according to the inclusion and exclusion criteria, including 60 type 1 patients (51 men and 9 women; mean age, 57.17 ± 12.17 years old) and 71 type 2 patients (55 men and 16 women; mean age, 58.56 ± 13.09 years old). The entire cohort of patients conforming to the inclusion criteria was divided randomly into the training set (n=91) and testing set (n=40) in a ratio of 7:3. The clinical factor data in the training and testing sets are shown in **Table 2**. Maximum diameter, shape, boundary, calcification, necrosis, renal vein invasion, lymph node metastasis and REV2 were statistically significant in differentiating pRCC type 1 and type 2 tumours after univariate analysis in the training set (both *p*<0.05). Multivariate logistic regression analyses were performed on the eight statistically significant clinical factors listed above. The *p*-value were 0.111, 0.770, 0.026, 0.342, 0.945, 1.000, 0.999 and 0.971 respectively. If the tumour boundary is blurred (OR, 2.352; 95%CI, 1.743-3.174), it is more likely to be a pRCC type 2 tumour.

**Table 2:** Clinical factors.

Clinical factors	Training cohort (n=91)		*p*	Testing cohort (n=40)		*p*
	Type1 (n=42)	Type2 (n=49)		Type1 (n=18)	Type2 (n=22)	
Gender			0.121			0.579
Male	35 (83%)	34 (69%)		16 (89%)	21 (95%)	
Female	7 (17%)	15 (31%)		2 (11%)	1 (5%)	
Age (years)	57.4 ± 12.2	58.8 ± 12.6	0.571	56.7 ± 12.4	58.1 ± 14.3	0.693
Maximum diameter (cm)	3.5 ± 2.0	6.0 ± 3.5	<0.001	3.6 ± 2.2	6.6 ± 2.7	0.001
Shape			0.001			<0.001
Round	38 (90%)	29 (59%)		17 (94%)	9 (41%)	
Not round	4(10%)	20 (41%)		1 (6%)	13 (59%)	
Location			0.174			0.356
Left	18 (43%)	28 (57%)		8 (44%)	13 (59%)	
Right	24 (57%)	21 (43%)		10 (56%)	9 (41%)	
Boundary			<0.001			0.004
Clear	41 (98%)	28 (57%)		16 (89%)	10 (45%)	
Blurred	1 (2%)	21 (43%)		2 (11%)	12 (55%)	
Calcification			0.004			0.427
Present	4 (10%)	17 (35%)		2 (11%)	5 (23%)	
Absent	38 (90%)	32 (65%)		16 (89%)	17 (77%)	
Necrosis			<0.001			<0.001
Present	6 (14%)	26 (53%)		2 (11%)	16 (73%)	
Absent	36 (86%)	23 (47%)		16 (89%)	6 (27%)	
Renal vein invasion			0.003			0.011
Present	0 (0)	9 (18%)		0 (0)	7 (32%)	
Absent	42 (100%)	40 (82%)		18 (100%)	15 (68%)	
Lymph node metastasis			0.001			0.005
Present	0 (0)	12 (24%)		0 (0)	8 (36%)	
Absent	42 (100%)	37 (76%)		18 (100%)	14 (64%)	
TEV1 (HU)	18.5 ± 17.4	27.4 ± 32.5	0.063	13.4 ± 7.3	29.8 ± 31.5	0.121
TEV2 (HU)	32.0 ± 21.3	38.6 ± 23.3	0.068	28.2 ± 11.4	43.1 ± 30.1	0.178
REV1	0.19 ± 0.16	0.28 ± 0.24	0.058	0.19 ± 0.20	0.30 ± 0.28	0.103
REV2	0.28 ± 0.28	0.33 ± 0.17	0.014	0.23 ± 0.12	0.42 ± 0.49	0.092

TEV, tumour enhancement value; REV, relative enhancement value; 1, corticomedullary phase; 2, nephrographic phase.

### Feature Extraction, Selection, and Radiomics Signature Construction

**Figure 4:**
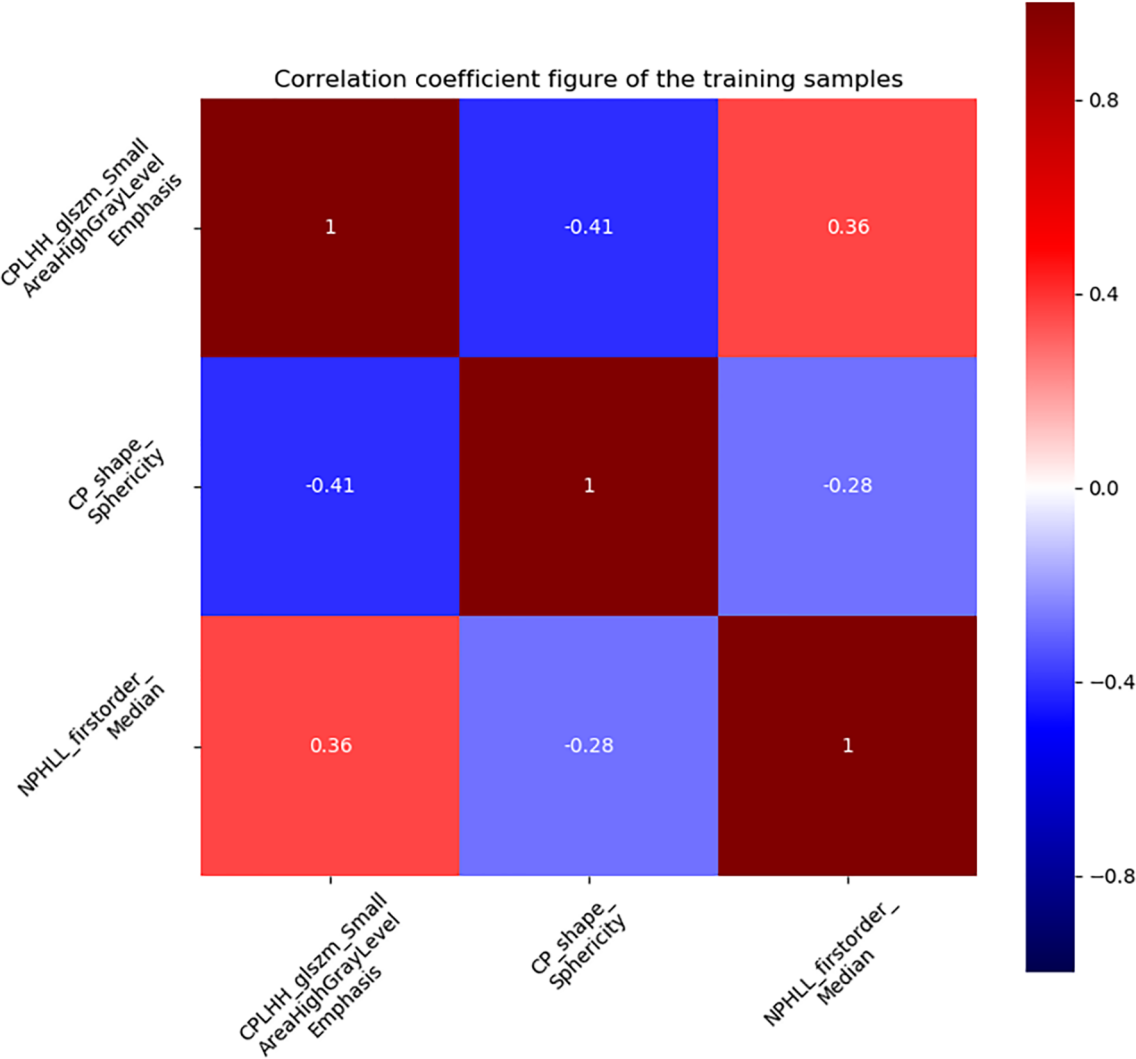
The correlation diagram of the three effective features screened out.

A total of 2632 radiomics features were extracted from the CMP and NP CT images, of which 1876 features had an ICCs greater than 0.75, indicating good inter-and intra- observer agreement for these features. By univariate correlation analysis, 282 radiomics features showed significant differences between type 1 and type 2 tumours. These features were sequentially imported into Gradient Boosting Decision Tree (21) and multivariate logistic analyses to obtain the most valuable features, resulting in three useful features (**Figure 4**). Finally, the radiomics signature was established by using three features. The AUC were 0.845 (95%CI 0.775-0.913) in the training set and 0.821 (95%CI 0.702-0.922) in the testing set. The Rad-score was calculated using the following formula:


$$\begin{array}{lll} \text{Rad}-\text{score}=-0.2964–1.1110\;\times \text{CMP}-\text{wavelet}-\text{LHH}\_\text{glszm}\_\text{SmallAreaHighGrayLevelEmpha} & +\;1.1637\times \text{CMP}-\text{original}\_\text{shape}\_\text{Sphericity} & +\;0.9529\times \text{NP}-\text{wavelet}-\text{HLL}\_\text{firstorder}\_\text{Median} \end{array}$$

**Figure 5:**
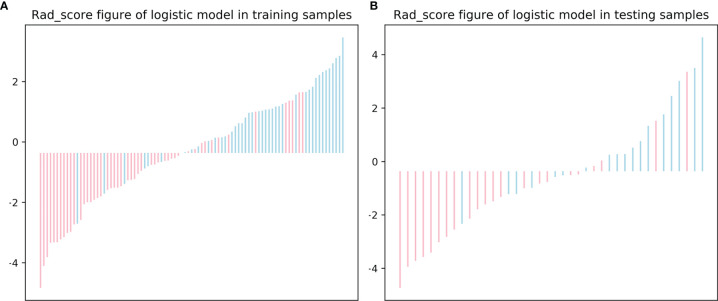
The radiomics score (Rad-score) for each patient in the training **(A)** and testing **(B)** sets.

The distribution of the Rad-score in the training and testing sets is shown in **Figure 5**.

### Establishment of Radiomics Nomogram and Evaluation of Performance Between Different Models

**Figure 6:**
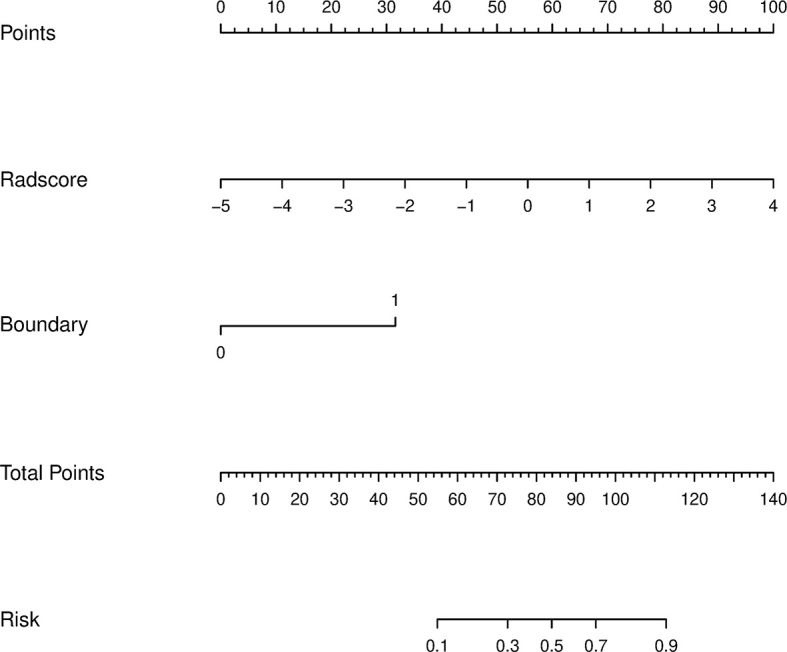
A radiomics nomogram distinguishing between type 1 and type 2 tumours. The nomogram was constructed by combining boundary and radiomics score (Rad-score) on the basis of a training cohort. The corresponding points are estimated from the boundary and Rad-score values, and these are added together to obtain total points. The likelihood of type 2 pRCC was estimated from the total points, the greater the total points, the greater the probability of type 2 pRCC.

**Figure 7:**
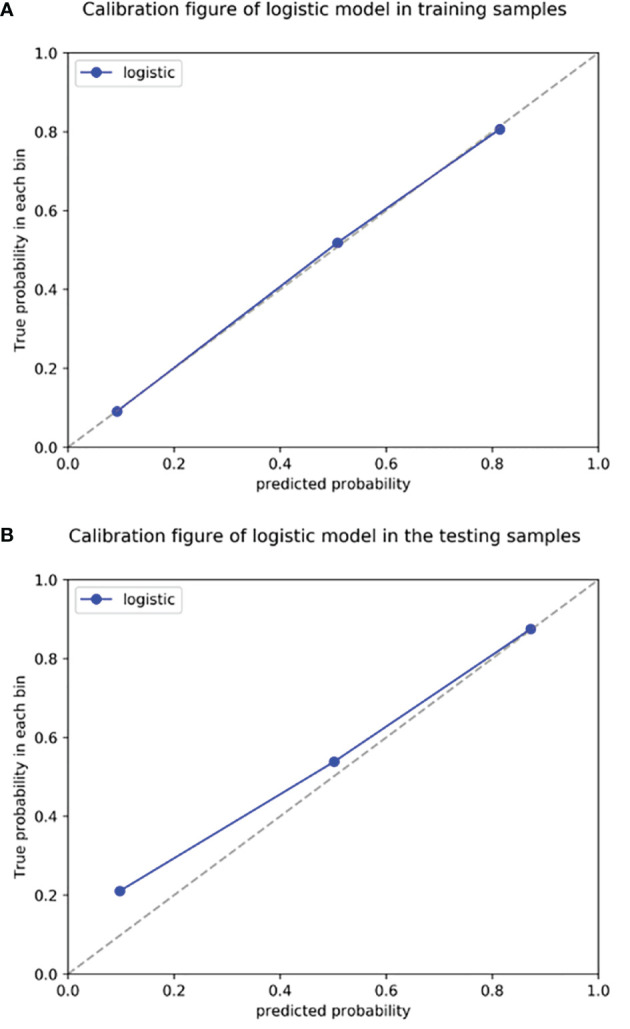
Radiomics nomogram calibration curves for the training **(A)** and testing **(B)** sets. The calibration curves show a good fit of the nomogram. The 45° straight lines indicate a perfect match between the true (Y-axis) and predicted (X-axis) probabilities. The closer the distance between the two curves, the better the accuracy.

**Figure 8:**
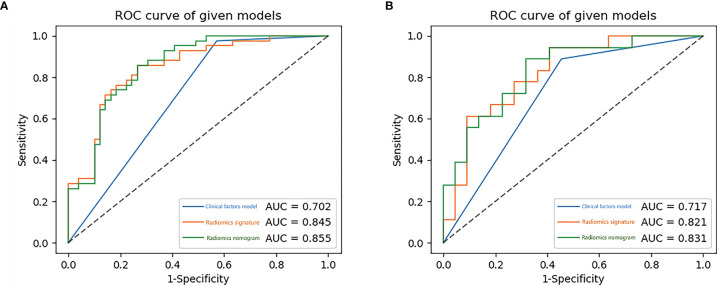
The receiver operating characteristic (ROC) curves of the clinical factors model, the radiomics signature and the radiomics nomogram for training **(A)** and testing **(B)** sets.

**Figure 9:**
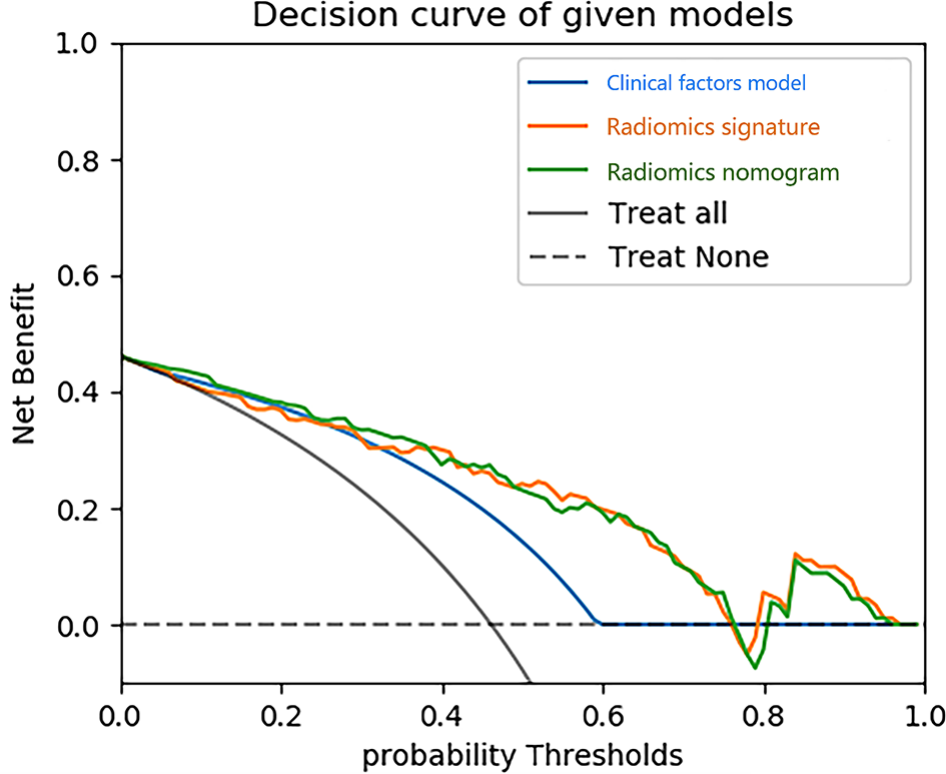
Decision curve analysis (DCA) for three models. The y-axis indicates the net benefit; x-axis indicates probability thresholds. The blue line, yellow line and green line represent net benefit of the clinical factors model, the radiomics signature and the radiomics nomogram, respectively. Both the radiomics nomogram and the radiomics signature showed a higher overall net benefit in differentiating type 1 from type 2 than the clinical factors model.

Using the data in the training set, a radiomics nomogram was established by combining important clinical factors which was the boundary information and Rad-score (**Figure 6**), and the radiomics nomogram score (Nomo-score) was calculated based on multivariate logistic regression analysis. The formula for calculating the Nomo-score for this study is shown below: Nomo-score = -2.1459 + B×2.3959 + R×0.8423 (B = Boundary; R = Rad-score). The calibration curves of the radiomics nomogram in **Figure 7** showed good calibration in both the training and testing cohorts. The discriminatory efficacies of the three diagnostic models (clinical factors model, radiomics signature and radiomics nomogram) are shown in **Table 3**. **Figure 8** plots the clinical factors model, radiomics signature and radiomics nomogram ROC curves based on the training cohort and testing cohort comparing the accuracies of these three models in identifying pRCC type 1 and type 2 tumours. The decision curves showed that in most training cohorts within reasonable threshold probabilities, the radiomics nomogram added greater overall net benefit in differentiating between pRCC type 1 and type 2 tumours compared to the clinical factors and radiomics signature. The DCA value for the three models in the training cohort are shown in **Figure 9**.

**Table 3:** Diagnostic performance of the clinical factors model, the radiomics signature and the radiomics nomogram.

Model	Training cohort		Testing cohort	
	AUC (95%CI)	Accuracy %	AUC (95%CI)	Accuracy %
Clinical factors model	0.702 (0.643,0.764)	53.8	0.717 (0.611,0.826)	55.0
Radiomics signature	0.845 (0.775,0.913)	78.0	0.821 (0.702,0.922)	75.0
Radiomics nomogram	0.855 (0.787,0.918)	78.0	0.831 (0.716,0.930)	75.0

AUC, area under the curve.

## Discussion

PRCC is the second most common subtype of RCC, second only to ccRCC. PRCC can be divided into two different subtypes, type 1 and type 2 (2). The systematic review and meta-analysis by Xiong et al. collected a total of 4494 pRCC patients from 22 studies and showed that overall survival and cancer specific survival was worse in type 2 pRCC patients than in type 1 pRCC patients (22). Because the two tumours have many differences in biology, treatment options and prognosis, it is clinically important to distinguish accurately between type 1 and type 2 tumours preoperatively. In the present study, the radiomics nomogram was constructed by combining clinical factors with Rad-score and was found to be highly accurate in distinguishing pRCC subtypes, with an AUC value of 0.855 in the training cohort.

Previous studies have shown that clinical and conventional CT and MRI images can help distinguish between pRCC type 1 and type 2 tumours (2, 8, 9, 23). Fourteen clinical factors were used for analysis in our study, mainly including gender, age, maximum diameter, shape, location, boundary, calcification, necrosis, renal vein invasion, lymph node metastasis, tumour enhancement value (TEV1 and TEV2) and relative enhancement value (REV1 and REV2). After multivariate logistic regression analysis, blurred tumour boundaries could be used as an independent predictor of type 2 tumours, which is in line with the results of previous studies (8–10). We believe that the most likely reason for this result is that type 2 tumours are highly malignant and aggressive, more likely to invade the fatty layer surrounding the kidney, resulting in poorly defined borders on CT images. In this study, type 2 tumours were significantly larger in diameter than type 1 tumours, and the difference was statistically significant (*p*<0.001), which is consistent with the findings of Egbert et al. (8) and Yamada et al. (9). In contrast, some of the findings showed that the difference in diameter between type 2 and type 1 tumours was not statistically significant (24–26). We found that type 2 tumours had more necrosis and calcification compared to type 1 tumours and that this difference was statistically significant, which is consistent with previous findings (8, 18, 27). Mydlo et al. (28) found that type 1 tumours were less enhanced than type 2 tumours on CT-enhanced scans, and we found no statistically significant difference between TEV1, REV1 in CMP and TEV2 in NP, while REV2 in NP type 2 tumours were significantly greater than type 1 tumours and the difference was statistically significant (*p*=0.014). We believe the reason for this outcome is the higher malignancy of type 2 tumours and the abundance of tumour neovascularization.

Radiomics is a newly emerging research method that has been widely used in the diagnosis and differential diagnosis of kidney tumours (17, 29–31). It aids clinical decision-making by extracting high-throughput quantitative data from images, thus enabling non-invasion analysis of tumour heterogeneity. Previous findings show that CT and MRI-based radiomics can be used to differentiate between pRCC type 1 and type 2 tumours. Wang et al. (32) collected 77 patients with RCC, including 32 ccRCC, 23 pRCC and 22 cRCC. The patients all underwent routine MRI (T2WI, EN-T1WI CMP, EN-T1WI NP) preoperatively, and a total of 39 radiomics features were extracted from the three sequences mentioned above. The final ROC curves were constructed and showed AUC values of 0.631-0.951 for differentiating ccRCC and cRCC; AUC values of 0.688-0.955 for differentiating pRCC and cRCC, and AUC values of 0.747-0.890 for differentiating ccRCC and pRCC. Yap et al. (33) extracted a total of 33 shape and 760 texture features from preoperative CT images of 735 renal masses (539 malignant and 196 benign) and used these features to build a radiomics model based on multiple machine learning classifiers for identifying benign and malignant renal masses. The AUC values were 0.64-0.68 for the shape features, 0.67-0.75 for the texture features, and 0.68-0.75 for the combination of shape and texture features. Nie et al. (30) collected a total of 99 patients who underwent preoperative CT examination and divided into a training set (n=80) and testing set (n=19) in order to construct a radiomics nomogram that could distinguish AML.wolf from hm-ccRCC preoperatively. A total of 14 valid features were selected from CMP and NP to build radiomics nomogram, which showed good discriminatory efficacy in both the training set (AUC, 0.896; 95%CI, 0.810-0.983) and the testing set (AUC, 0.949; 95%CI, 0.856-1.000). Its discriminatory power was higher than that of the clinical factors model and the radiomics signature. Doshi et al. (10) assessed whether qualitative features (signal intensity, heterogeneity, and margin) and quantitative textural features (ADC, HASTE, and contrast-enhanced entropy) from preoperative MRI images of 21 pRCC type 1 tumours and 17 type 2 tumours could be for preoperative differentiation between type 1 and type 2 tumours. The results showed that the AUC values were 0.822 for the qualitative feature model, 0.682-0.716 for the quantitative feature model, and 0.859 for the combined qualitative and quantitative feature model. Duan et al. (17) extracted textures features based on 62 preoperative three-phase enhanced CT images of pRCC (30 type 1 tumours and 32 type 2 tumours) and built a model based on an SVM classifier. The AUC values were 0.772-0.753 for the CMP-based model, 0.832-0.841 for the NP-based model, 0.849-0.858 for the EP-based model, and 0.922 for the combined three-phase model. The results showed that CT-based texture analysis could be used to preoperatively differentiate between type 1 and type 2 tumours.

The nomogram is a practical and straightforward statistical prediction tool that has been widely used to combine multiple risk factors to predict medical prognosis and outcomes (34). Huang et al. (35) combined clinical factors with radiomics signature to construct a nomogram for predicting disease-free survival in non-small lung cancer. The nomogram’s diagnostic efficacy was higher than clinical factors alone. Our study builds a nomogram based on boundary and Rad-score to predict the probability of type 1 tumours with AUC values of 0.855, 0.831 in the training and testing sets, respectively. The AUC values for the model constructed on clinical factors alone were 0.702,0.717 in the training and testing sets, respectively. The high diagnostic efficacy of the nomogram over clinical factors alone suggests that the Rad-score is of high value in differentiating between type 1 and type 2 tumours.

Compared to the above radiomics studies, our study had some differences and provided some improvements: First, our study focused on distinguishing between type 1 pRCC and type 2 pRCC, mainly because type 1 and type 2 tumours often have many overlapping imaging presentations in CT images. Second, a total of three radiomics features were extracted from the CMP and NP images, two of which were derived from the CMP images, indicating that the CMP images have higher diagnostic efficacy in differentiating type 1 and type 2 tumours. Third, this study combined clinical factors with radiomics features for the construction of the model, enabling a more comprehensive assessment of tumour characteristics and allowing more reliable results to be obtained. Fourth, most previous studies tend to base their texture analysis on one dimension of the tumour, whereas we mainly used all dimensions of the tumour to analyse the tumour and obtain more features. While previous studies mainly extracted a few dozen features, we extracted over 1000 features. Finally, although pRCC is a relatively rare type of RCC, a total of 131 cases of pRCC were collected in our study. To our knowledge, this is the largest sample size to date to study a radiomics-based subtype of pRCC, and our sample was derived from multiple centres.

There are several limitations to our current study. First, this study was a retrospective study, which may introduce bias in the selection of the sample and overestimation of diagnostic accuracy, so external validation may be included in subsequent studies. Second, our study only extracted radiomics features from the CMP and NP images for tumour analysis, and in the future more features may be extracted from the four-phase images of CT to obtain more radiomics information of the tumour. Third, in this study, we used a variety of CT scanners from different suppliers, and although we have normalized the images before extracting the features, there is still the potential for error in the experiment. Fourth, manual segmentation of 3D ROI is both time-consuming and complicated, especially for tumours with unclear borders. Further research should focus on developing an automatic segmentation method for renal tumours with better reliability and reproducibility. Final, the primary target of this study was pRCC and did not include other types of renal tumours. In subsequent studies, we will collect more cases to build a complete model for differentiating subtypes of renal tumours.

In conclusion, our study demonstrates the importance of combining clinical factors with radiomics features to construct a CT-based radiomics nomogram of CMP and NP images. Our radiomics nomogram can distinguish between pRCC type 1 and type 2 tumours preoperatively and has good diagnostic performance. As a new non-invasion, quantitative diagnostic method, the use of radiomics nomogram needs further validation before it can be used in the clinic.

## Data Availability Statement

The original contributions presented in the study are included in the article/supplementary material. Further inquiries can be directed to the corresponding author.

## Ethics Statement

The studies involving human participants were reviewed and approved by The First Affiliated Hospital of Anhui Medical University. The ethics committee waived the requirement of written informed consent for participation.

## Author Contributions

YG: The acquisition of data, analysis of data, and drafting the article. XWWa, SW, YM, CZ, CL and GH: The acquisition of data. YJ: The data analysis. JL: The analysis and interpretation of data. XZ: Drafting and revision of manuscripts. XWWu: Final approval of the version to be submitted. All authors contributed to the article and approved the submitted version.

## Funding

This work was supported by 2021 Medical Empowerment- Pilot Elite Research Project Special Fund (NO. XM_HR_YXFN_2021_05_19).

## Conflict of Interest

Author JL was employed by GE Healthcare China.

The remaining authors declare that the research was conducted in the absence of any commercial or financial relationships that could be construed as a potential conflict of interest.

## Publisher’s Note

All claims expressed in this article are solely those of the authors and do not necessarily represent those of their affiliated organizations, or those of the publisher, the editors and the reviewers. Any product that may be evaluated in this article, or claim that may be made by its manufacturer, is not guaranteed or endorsed by the publisher.
